# Nasal high flow clears anatomical dead space in upper airway models

**DOI:** 10.1152/japplphysiol.00934.2014

**Published:** 2015-04-16

**Authors:** Winfried Möller, Gülnaz Celik, Sheng Feng, Peter Bartenstein, Gabriele Meyer, Oliver Eickelberg, Otmar Schmid, Stanislav Tatkov

**Affiliations:** ^1^Comprehensive Pneumology Center, Member of the German Center for Lung Research, Munich, Germany;; ^2^Institute of Lung Biology and Disease, Helmholtz Zentrum München, German Research Center for Environmental Health, Neuherberg, Germany;; ^3^Fisher and Paykel Healthcare, Auckland, New Zealand;; ^4^Department of Nuclear Medicine, LMU Medical Center Grosshadern, München, Germany;; ^5^Asklepios Fachkliniken München-Gauting, Department of Nuclear Medicine, Gauting, Germany; and; ^6^University Hospital, Ludwig-Maximilians-University, Munich, Germany

**Keywords:** nasal high flow, insufflation, upper airways, dead space, carbon dioxide, krypton

## Abstract

Recent studies showed that nasal high flow (NHF) with or without supplemental oxygen can assist ventilation of patients with chronic respiratory and sleep disorders. The hypothesis of this study was to test whether NHF can clear dead space in two different models of the upper nasal airways. The first was a simple tube model consisting of a nozzle to simulate the nasal valve area, connected to a cylindrical tube to simulate the nasal cavity. The second was a more complex anatomically representative upper airway model, constructed from segmented CT-scan images of a healthy volunteer. After filling the models with tracer gases, NHF was delivered at rates of 15, 30, and 45 l/min. The tracer gas clearance was determined using dynamic infrared CO_2_ spectroscopy and ^81m^Kr-gas radioactive gamma camera imaging. There was a similar tracer-gas clearance characteristic in the tube model and the upper airway model: clearance half-times were below 1.0 s and decreased with increasing NHF rates. For both models, the anterior compartments demonstrated faster clearance levels (half-times < 0.5 s) and the posterior sections showed slower clearance (half-times < 1.0 s). Both imaging methods showed similar flow-dependent tracer-gas clearance in the models. For the anatomically based model, there was complete tracer-gas removal from the nasal cavities within 1.0 s. The level of clearance in the nasal cavities increased by 1.8 ml/s for every 1.0 l/min increase in the rate of NHF. The study has demonstrated the fast-occurring clearance of nasal cavities by NHF therapy, which is capable of reducing of dead space rebreathing.

respiratory failure is a common complication in a range of pulmonary conditions (12). Recent studies report that an open nasal cannula system for delivering of nasal high flow (NHF) can assist ventilation in patients with chronic respiratory failure (2, 3, 5, 10) and sleep disorders (14, 18). The concept of delivering high flow through the open nasal cannula is not entirely new (24), but advancements in technology that efficiently warm and humidify respiratory gases have been the key factor for clinical application of NHF. This form of respiratory support is commonly used with a wide range of flow from 2 l/min in preterm newborns to 60 l/min in adults with or without supplemental oxygen (7, 25). NHF can also be combined with a delivery of aerosolized drugs into the airways (1, 4).

A number of clinically relevant benefits have been associated with NHF therapy: reduction in respiratory rate, an increase or decrease in minute ventilation, improved alveolar ventilation, and a reduction of wasted ventilation and the work of breathing (5, 10). However, the mechanisms of how NHF produces these benefits are poorly understood. A mechanistic study of NHF proposed two different ventilatory responses, one when awake and another during sleep (16). The reduction of dead-space ventilation was proposed to be the principal driver for the response during sleep. The mechanisms of dead-space clearance are difficult to study due to the anatomical complexity and inability to visualize the gas flow in the upper airways. However, many researchers have proposed dead-space clearance during NHF as the major physiological mechanism that improves respiratory support (17, 20, 22). Measurement of carbon dioxide (CO_2_) concentration in the trachea confirmed this hypothesis (21), and other studies have also reported a reduction of arterial and tissue CO_2_ in response to NHF therapy (3, 6).

In this study, the clearance of gas in dead space with NHF was investigated using two upper airway models. The first was a simple tube model (TM), which consisted of a nozzle and a cylindrical tube. The nozzle represented the nasal valve area, which is the narrowest constriction of the upper airways, and the tube characterized the volume of the upper airways. The second was an upper airway model (UAM), which more accurately represented the complexity of the upper airways. For the simple TM, the gas clearance rates were quantified using both a midwave infrared (MWIR) CO_2_ absorption spectroscopy and by radioactive krypton (^81m^Kr-gas) gamma camera imaging. For the more anatomically accurate UAM, only the gamma camera imaging could be used due to the materials available for 3D printing being incompatible with MWIR spectroscopy.

The main hypothesis of this study was to experimentally demonstrate that NHF can clear tracer gases from upper airway models independent of the tracer gas, the imaging modality, or the dimensional complexity of the models tested. It was also hypothesized that NHF flow rates would be a major factor in the level of clearance, with the anatomical complexity and interindividual variability in the anatomy of the upper airways playing less important roles.

## METHODS

### 

#### Nasal high flow.

**Fig. 1.**
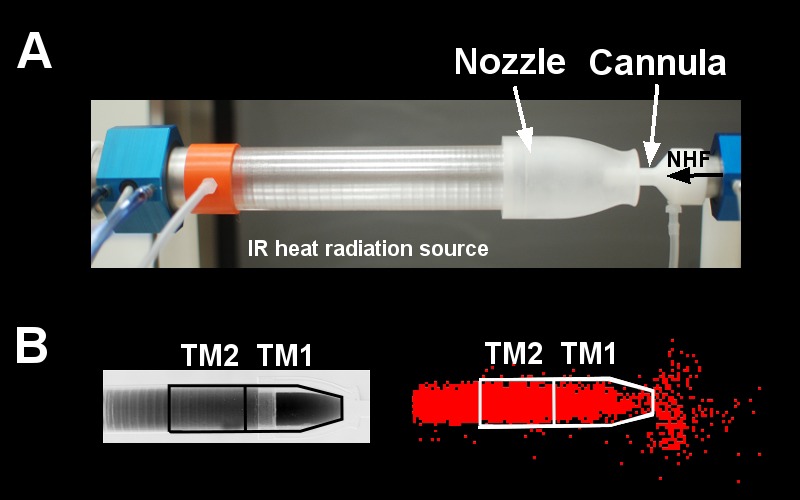
*A*: upper airway tube model (TM) made from a sapphire tube and a sodium chloride (NaCl) nozzle with a cannula inserted into the nozzle in front of the IR-heat radiation source (blackbody). Also shown are the pressure ports and the pneumotachographs to monitor pressure and flow within the tube and the cannula. The cannula flow rates [nasal high flow (NHF)] were delivered into the nozzle at 15, 30, and 45 l/min. *B*: an infrared absorption image (left) and a gamma camera image (right) show the filling stage of the model with CO_2_ and ^81m^Kr gas before air was flushed into the cannula. Anterior (TM1) and posterior (TM2) ROIs were defined for data analysis.

Nasal high flow (NHF) rates of 15, 30, and 45 l/min of air were generated using a high-flow blower-humidifier (AIRVO 2, Fisher and Paykel Healthcare, New Zealand). The delivered flow was measured by a low-resistance pneumotachograph (Fleisch, Lausanne, Switzerland) and a differential pressure transducer carrier-amplifier system (Validyne, Northridge, CA). The high-flow blower-humidifier was always on, to allow the system to be at stable operational temperatures and flow rates. A valve (Rudolph, Shawnee, KS) is used to alternate between two cannulas, one delivering ^81m^Kr-gas for model filling and the other delivering NHF for clearance of ^81m^Kr-gas. When measurements were taken, the tracer gas was introduced into the model and then the Y-valve directed NHF through the cannula. For the simplified TM experiments (Fig. 1*A*), a custom-made cannula (ID = 6.3 mm, OD = 7.0 mm, length = 40.0 mm) was used to deliver the NHF, while for the UAM experiments an Optiflow cannula interface (OPT844, Fisher and Paykel Healthcare, New Zealand) was used.

Two upper airway models and two different imaging systems were used in this study. The first model was a simple tube model (TM) geometry that allowed both imaging systems to visualize the gas clearance, and a second one was a more complicated upper airway model (UAM) that only allowed the radioactive ^81m^Kr-gas to be visualized. Therefore, the TM was used to compare the imaging systems with different gas compositions, while the second imaging system enabled visualization in the more anatomically accurate geometry. CO_2_ MWIR absorption spectroscopy and ^81m^Kr-gas radioactive gamma camera imaging were used as imaging systems in this study. The first imaging system used Carbogen, which better represented the expired gases, and the second used air labeled with ^81m^Kr-gas, which could also be imaged through the more complex upper airway model.

#### MWIR absorption spectroscopy.

The first imaging system used MWIR absorption spectroscopy to visualize CO_2_ tracer-gas clearance. It comprised a custom-made blackbody (Fig. 1*A*), controlled to 230°C, to be used as the heat radiation source, and a MWIR camera system (SC7600, FLIR, France), together with a narrow band-pass filter 4,260/20 nm (Spectrogon, Sweden). The CO_2_-filled TM was placed between the IR heat source and the MWIR camera system (Fig. 1*A*), which detected heat radiation absorption by CO_2_ in the tube. A Carbogen gas mixture of similar composition to expired air (6% CO_2_, 21% O_2_, and 73% N_2_; BOC Gases, Auckland, New Zealand) was used as a tracer gas to closely represent gas behavior during NHF therapy.

#### ^81m^Kr tracer-gas imaging.

The second imaging system used a planar gamma camera (Orbiter; Siemens, Erlangen, Germany) to visualize radioactive ^81m^Kr tracer-gas clearance. For these experiments, the ^81m^Kr-gas (TycoHC Covidien, Neustadt, Germany) was generated at a concentration of 1-2% in the entrained air. The models filled with the ^81m^Kr tracer gas were placed in front of the planar gamma camera. The gamma camera sampled images at a 25-Hz frame rate. The clearance curves were then fitted with exponential functions and the clearance half-times calculated. These data were corrected to allow for the level of natural ^81m^Kr-gas decay (T_1/2_ = 13 s) and presented as corrected clearance half-times.

#### Tube model (TM).

The simplified geometry of the TM had two distinct compartments. The first was a single nozzle representing the combined nasal valve area (ID of 12 mm) of both noses, machined from a single sodium chloride (NaCl) crystal. Directly coupled to the nozzle was the second tube compartment, which represented the volume of the upper airways. This was fabricated from a grown sapphire crystal tube of dimension ID = 26 mm, OD = 31 mm, length = 130 mm (Fig. 1*A*). This model was used for both CO_2_ absorption spectroscopy imaging and ^81m^Kr tracer-gas X-ray imaging. Both the NaCl and sapphire compartments exhibited excellent transmission characteristics in the MWIR spectrum, allowing high-efficiency CO_2_ imaging within the 4.26-μm absorption band, as well as the ^81m^Kr tracer-gas imaging. For both tracer gases, the TM was filled with the tracer gas from the side opposite to the nozzle. Then the administration of NHF was carried out via a cannula, which was placed coaxially in the nozzle of the TM (Fig. 1*A*). The induced dilution and wash-out of the tracer gases, or dead-space clearance, was recorded by the appropriate imaging system, allowing direct comparison of clearance rates within the TM.

As illustrated in Fig. 1*B*, two regions of interest (ROIs) were defined in the simple TM, those being the anterior section of the nozzle (TM1, volume 28 cm^3^) and posterior part of the nozzle (TM2, volume 25 cm^3^). CO_2_ clearance profiles for these ROIs were analyzed and characterized by fitting exponentially decaying functions. The CO_2_ clearance half-times for these ROIs were then calculated. For the experiments using the ^81m^Kr-gas imaging system with the simplified TM, images were captured for both the ^81m^Kr-gas filling and subsequent 15 s of NHF clearance. Similar ROIs to those used in the CO_2_ imaging were applied to analyze the ^81m^Kr-gas clearance characteristics from the TM (Fig. 1*B*). Clearance rates were determined from the ROI volumes divided by the appropriate clearance time constants.

**Fig. 2.**
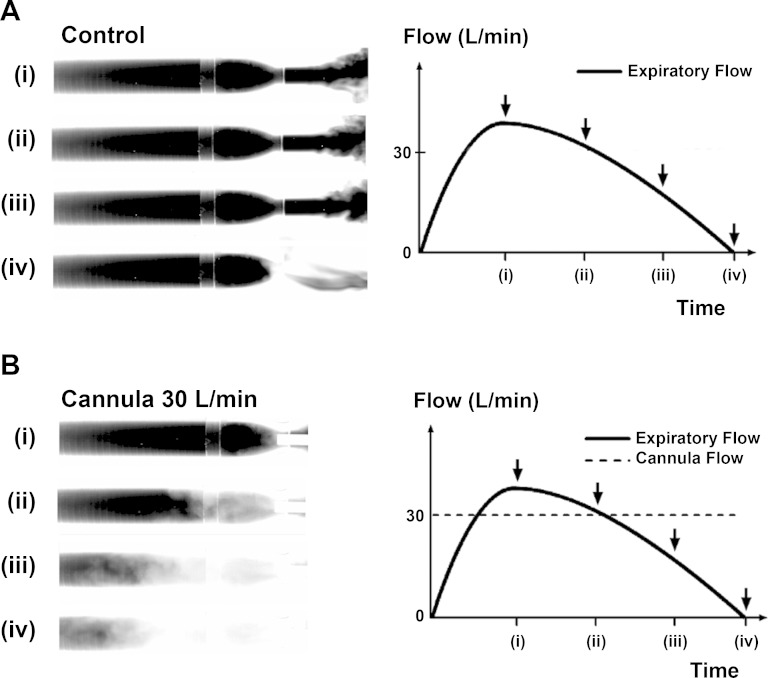
Infrared absorption images of expiratory flow through a tube model (TM) of upper airways demonstrate rebreathing from dead space. The images show four stages of filling of the model with exhaled CO_2_ at (*i*) peak expiratory flow, (*ii*) expiratory flow 30 l/min, (*iii*) expiratory flow 15 l/min, and (*iv*) end of expiration. *A*: control demonstrates filling of the TM during the expiration phase without NHF from a cannula. At the beginning of inspiration all gas from the TM will be rebreathed into the lungs. *B*: NHF from the cannula purges the expired CO_2_-rich gas from the model and replaces it with fresh air. This results in a reduction of CO_2_ rebreathing. Breathing through the model demonstrates that the replacement of expired gas with air starts before the end of expiration and that the static conditions used in the experiments led to an underestimation of the speed of dead-space clearance during respiration.

Figure 2*A* demonstrates exhalation flow profiles through the TM and visualization of expired CO_2_. Figure 2*B* shows clearance of CO_2_ in the TM during 30 l/min flow through the cannula into the nozzle.

#### Upper airway model.

**Fig. 3.**
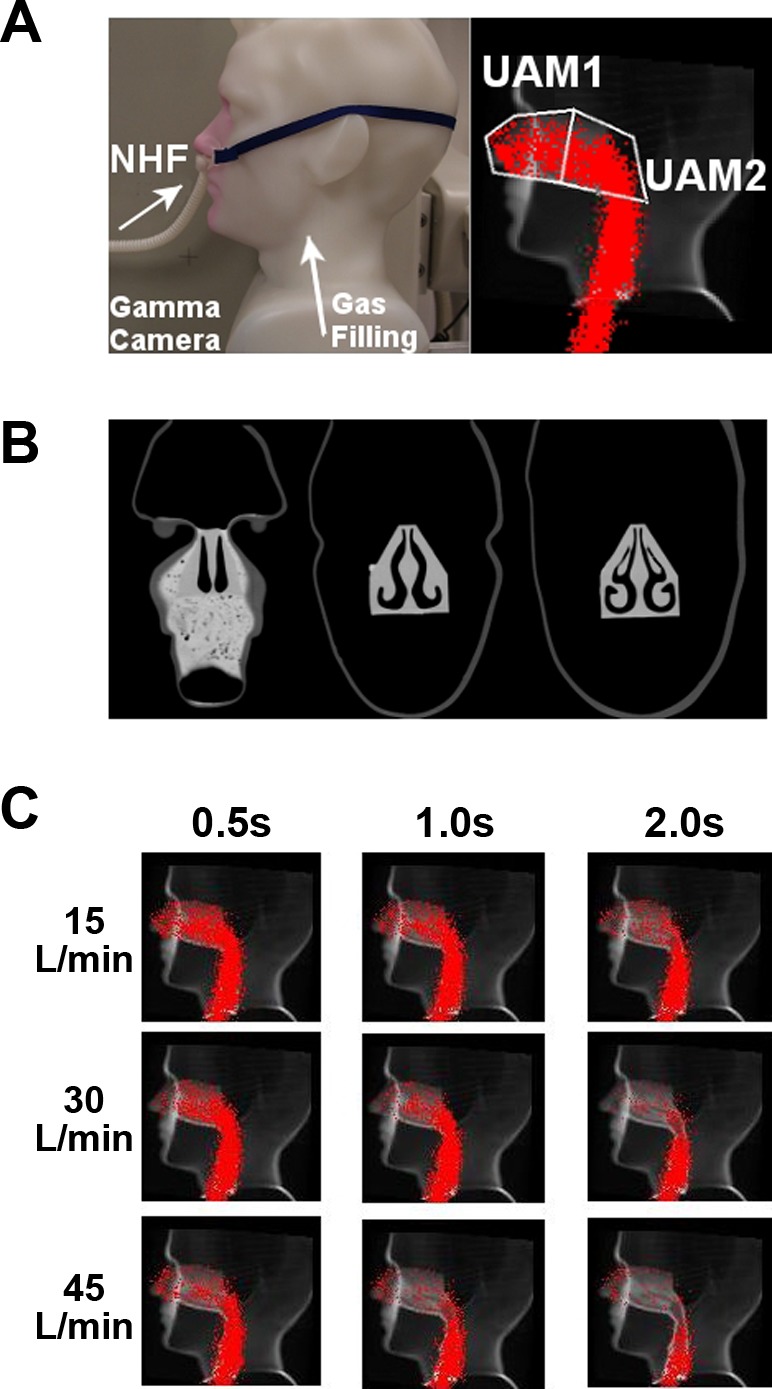
*A, left*: standard image of the upper airway model (UAM) showing the setup of the cannula interface (left panel) in the nostrils. *A, right*: the same image overlaid with the outlines of the anterior (UAM1) and posterior (UAM2) ROIs in the nasal cavities, and data from the planar gamma camera when the UAM was filled from the trachea end with ^81m^Kr-gas. *B*: coronary CT scans of the model, illustrating the complex internal anatomical structure in the UAM. *C*: lateral gamma camera images of ^81m^Kr-gas filling of UAM superimposed onto a sagittal CT of the UAM. Series of images illustrate the tracer-gas clearance at time points 0.5, 1.0, and 2.0 s using NHF rates 15, 30, and 45 l/min.

An anatomically accurate 3D upper airway model (UAM) was developed to better represent the expected gas clearance when using NHF therapy in practice (Fig. 3*A*). The UAM was based on segmented images from a computer tomography (CT) scan of a healthy volunteer, which was then constructed using a High Definition (HD) 3D printer (Projet HD3000, 3D Systems). Nasal valve area in both noses was 56 mm^2^. There was no anatomical structure beyond the nasal cavity, and the unit led into an 18-mm ID tubing, which exited at the bottom of the model. The material in the 3D printer is highly absorbent in the MWIR spectrum. Therefore, only the ^81m^Kr-gas gamma camera imaging was used. The UAM was then integrated into a plastic head model to enable the attachment of the cannula interface. The position of the UAM in front of the gamma camera and the attachment of the NHF cannula interface are shown in Fig. 3*A* (left panel). The anterior and posterior ROIs (UAM1 and UAM2, respectively), illustrated in Fig. 3*A* (*right*), were overlaid with a CT image of the UAM (Fig. 3*B*) showing the detailed anatomical accurate nasal cavity structures contained within the model. For this protocol, the UAM was filled with ^81m^Kr-gas, via the tubing from bottom, while the nasal cannula was located in the nares, in line with normal use. As for the TM experiments, both the filling and 15 s of ^81m^Kr-gas clearance were captured by dynamic gamma camera imaging.

Dynamic ^81m^Kr-gas activity profiles were constructed from the sagittal plane gamma camera imaging. For identification of anatomical markers in the gamma camera recordings, all serial images were superimposed and overlaid on a representative CT slice of the UAM, and ROIs were defined (Fig. 3*A*) in the nasal cavities, which were divided into anterior (UAM1) and posterior (UAM2) ROIs. Clearance rates were calculated using the total volume (55 cm^3^) of the nasal cavities.

#### Data analysis.

Every experimental condition was repeated five times. Statistical analyses were performed using Winstat 2009.1 (Microsoft Excel 2009), to determine the mean ± SD, median, minimum, and maximum values. Differences between each experimental condition were compared using a two-sided *t*-test with a significance level of *P* < 0.05. In addition, differences were assessed using a paired *t*-test with the same significance level. A Pearson correlation analysis was performed to assess correlations among study variables.

## RESULTS

### 

#### CO_2_- and ^81m^Kr-gas clearance in the TM.

**Fig. 4.**
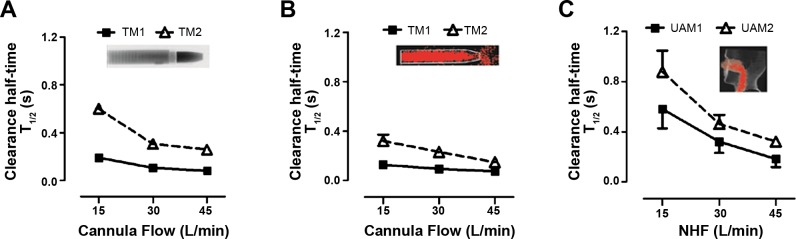
Comparison of clearance profiles during flow rates of 15, 30, and 45 l/min from a custom-made cannula in the TM and a standard cannula interface in the UAM model using comparable ROIs. *A*: clearance half-time (T_1/2_) in the TM with CO_2_-gas MWIR imaging experiments. *B*: clearance half-time (T_1/2_) in the TM with ^81m^Kr-gas gamma imaging experiments. *C*: clearance half-time (T_1/2_) in the UAM with ^81m^Kr-gas gamma imaging experiments. The clearance profiles are similar in all three experiments. Anterior ROIs (TM1 and UAM1) are cleared faster than posterior ROIs (TM2 and UAM2). Clearance in posterior ROIs is more flow dependent than in anterior ROIs.

The clearance half-times for the two ROIs vs. the NHF rates for the CO_2_ clearance experiments are shown in Table 1 and plotted in Fig. 4*A*. The comparable clearance half-times of ^81m^Kr-gas are shown in Table 2 and in Fig. 4*B*. For either ROI in the TM, all NHF rates and both imaging techniques had clearance half-times of 0.6 s or less. For all flow rates and both tracer gases, the anterior ROI (TM1) clearance half-time was always faster than the posterior ROI (TM2) (*P* < 0.01). For both tracer gases and ROIs, the clearance half-times decreased with increasing NHF rates (*r* = −0.84, *P* < 0.001). At 45 l/min NHF, the clearance half-time was approximately half that for an NHF of 15 l/min. The clearance half-times for the CO_2_ experiments demonstrated a highly positive correlation with the ^81m^Kr-gas clearance rates (*r* = 0.97, *P* < 0.001) for both ROIs.

**Table 1.** Half-times (T_1/2_) of CO_2_-gas clearance in the proximal (TM1) and medium (TM2) ROIs of the tube model during flow from a cannula for NHF rates of 15, 30, and 45 l/min

	Cannula Flow		
	15 l/min	30 l/min	45 l/min
TM1: T_1/2_, s	0.19 ± 0.01	0.11 ± 0.01*	0.08 ± 0.01*
Median (min, max)	0.2 (0.2, 0.2)	0.1 (0.1, 0.1)	0.1 (0.1, 0.1)
TM2: T_1/2_, s	0.60 ± 0.04†	0.31 ± 0.03*†	0.26 ± 0.01*†
Median (min, max)	0.6 (0.5, 0.6)	0.3 (0.3, 0.4)	0.3 (0.2, 0.3)

Values are means ± SD, median, minimum, and maximum. TM, tube model; ROI, region of interest; NHF, nasal high flow.

* *P* < 0.01 compared with15 l/min NHF;

† *P* < 0.01 for TM2 compared with TM1.

**Table 2.** Half-times (T_1/2_) of ^81m^Kr-gas clearance in the proximal (TM1) and medium (TM2) ROIs of the tube model during flow from a nasal cannula for NHF rates of 15, 30, and 45 l/min

	Cannula Flow		
	15 l/min	30 l/min	45 l/min
TM1: T_1/2_, s	0.13 ± 0.02	0.09 ± 0.01*	0.07 ± 0.01*
Median (min, max)	0.1 (0.1, 0.1)	0.1 (0.1, 0.1)	0.1 (0.1, 0.1)
TM2: T_1/2_, s	0.32 ± 0.05+	0.23 ± 0.03*†	0.15 ± 0.01*†
Median (min, max)	0.3 (0.3, 0.4)	0.2 (0.2, 0.2)	0.1 (0.1, 0.2)

Values are means ± SD, median, minimum, and maximum.

* *P* < 0.01 compared with15 l/min NHF;

† *P* < 0.01 for TM2 compared with TM1.

Tracer-gas (CO_2_) wash-out during exhalation through the TM and application of 30 l/min cannula flow revealed the onset of tracer-gas clearance early, before the end of exhalation (Fig. 2). It is effective when the exhalation flow decreases below the cannula flow (time *point ii* in Fig. 2).

#### ^81m^Kr-gas clearance in the UAM.

Typical examples of the data from the UAM using the gamma camera can be seen in Fig. 3*C* and the Supplemental Video available with the online version of this article. The UAM was filled with ^81m^Kr gas, from the trachea end, and NHF rates of 15, 30, and 45 l/min were introduced through a nasal cannula interface. The images shown in Fig. 3*C* were obtained from the dynamic stack and represent the tracer-gas distribution at three time periods (0.5, 1.0, and 2.0 s) following the introduction of NHF. The images show the rapid clearance of the ^81m^Kr-gas from the nasal cavity 0.5 s after the onset of NHF, and deeper clearance was associated with greater NHF rates and longer times.

The ^81m^Kr-gas clearance half-times observed in the UAM at the NHF rates tested are summarized in Table 3 and illustrated in Fig. 4*C*. The characteristic dependencies of gas clearance from the UAM are comparable to those during use of the TM. Clearance half-time decreases by increasing the NHF rate (*r* = −0.84, *P* < 0.001). For both the TM and UAM experiments, the anterior ROI (TM1 and UAM1, respectively) demonstrated the fastest clearance rates (*P* < 0.01), and the clearance half-times decreased by increasing the NHF rate for both ROIs. In addition, clearance half-times of ^81m^Kr-gas from the UAM's ROIs correlate with the comparable TM's ROIs (*r* = 0.95, *P* < 0.001).

**Table 3.** Half-times (T_1/2_) of ^81m^Kr-gas clearance in the anterior (UAM1) and posterior (UAM2) ROIs of the nasal cavity of the upper airway model for NHF rates of 15, 30, and 45 l/min

	NHF		
	15 l/min	30 l/min	45 l/min
UAM1, T_1/2_, s	0.58 ± 0.15	0.32 ± 0.09*	0.18 ± 0.06*
Median (min, max)	0.6 (0.4, 0.8)	0.3 (0.2, 0.4)	0.2 (0.1, 0.3)
UAM2, T_1/2_, s	0.88 ± 0.17†	0.46 ± 0.07*†	0.32 ± 0.02*†
Median (min, max)	0.9 (0.7, 1.1)	0.5 (0.4, 0.6)	0.3 (0.3, 0.4)

Values are means ± SD, median, minimum, and maximum. UAM, upper airway model.

* *P* < 0.01 compared with15 l/min NHF;

† *P* < 0.01 for UAM2 compared with UAM1.

**Fig. 5.**
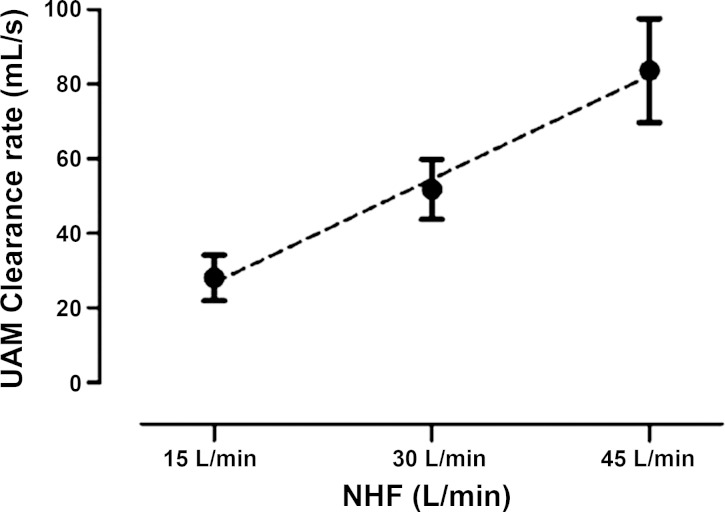
Clearance rates in nasal cavities (total volume 55 ml) of the upper airway model (UAM) at NHF rates of 15, 30, and 45 l/min, calculated from the clearance half-times and corresponding volumes of UAM1 and UAM2 ROIs. The clearance rate linearly rises with an increase of NHF. The graph shows that in the static experimental setup NHF of 30 l/min clears the total volume of the nasal cavity within 1 s.

The total clearance rate from both UAM nasal cavity ROIs expressed in milliliters per second (Fig. 5) has a linear relationship with the NHF rates (15, 30, and 45 l/min) tested (*r* = 0.92, *P* < 0.001): every 1 l/min increase in NHF results in 1.8 ml/s increased clearance in the nasal cavities.

## DISCUSSION

Recent studies have reported clinical benefits of NHF (3, 15, 18), with the key mechanism being hypothesized as the clearance of dead space resulting in a reduction of CO_2_ rebreathing (5, 13). However, dead-space clearance is very difficult to study in vivo due to the challenges of quantifying this rapid process in an anatomically complex environment. Animal models are of limited use because of their significant differences in the anatomy of their upper airways. In this study, the dead-space clearance rates for two very geometrically different models were studied. The effects of breathing on dead-space clearance were excluded in order to guarantee reproducible experimental conditions. Therefore, the studies were performed to simulate quasi-static breath-holding conditions. The tracer gases had been introduced into the models before NHF was commenced, to record sequences of images.

The first tube model (TM) represented a simplified airway for the ease of clearance, but allowed both tracer gases to be imaged in a consistent framework. A single nozzle represented the two nasal valve areas, which are the narrowest part in upper airways. A cannula with thin walls was positioned in the center of the nozzle and a tube behind the nozzle represented the dead-space volume in conducting airways. This was a similar airway model to that used in an earlier study to demonstrate the pressure/flow relationship during NHF therapy (16). The airway model was filled with either CO_2_ or ^81m^Kr tracer gas, and then the wash-out and clearance characteristics caused by the delivery of high-flow air via the cannula were quantified using either dynamic MWIR or radioactive gamma camera imaging, respectively. Spectroscopic imaging of the Carbogen gas mixture within the TM allowed visualization of the gas flow at 1,000 frames/s. This was then postprocessed to allow quantification of the gas clearance rates, as CO_2_ is a key component of the expired gas and imaging this gas is physiologically relevant. However, only a small number of materials that are very difficult to manufacture are transparent in the MWIR spectrum. This limited the complexity of the upper airway model to a simple tube, with a valve region. Therefore, the technique of gamma camera imaging of ^81m^Kr-gas was used, as this allowed much more complex upper airway models to be imaged from a wider range of materials. One weakness of the gamma camera imaging was that it had lower spatial and temporal resolution than the MWIR spectroscopy. Gamma camera imaging can also be used in vivo to visualize the airways in healthy volunteers because krypton is an inert noble gas and the isotope ^81m^Kr produces a very low radiation dose.

The results from both the CO_2_ and ^81m^Kr-gas imaging have demonstrated very fast clearance of the tracer gas following the application of high flow through the cannula. The clearance half-times in the simple TM nasal cavity were less than 0.6 s for both tracer gases. There was a similar flow-rate dependency of the compartmental clearance half-time for both tracer gases, and higher flow rates significantly reduced the clearance half-times. The gases leaked around the cannula and the clearance profiles suggest two specific characteristics: *1*) for all flows studied, clearance is faster in the anterior ROI and slower in the posterior ROI; and *2*) clearance half-time decreases as the NHF rate increases for all ROIs. The strong correlation between the data from both gases in the TM experiments shows that the two imaging systems are comparable. The significantly slower acquisition rate of the ^81m^Kr-gas gamma camera imaging system can still be used to study kinetics of these very rapid processes in more complex anatomically realistic models where CO_2_ visualization is inhibited. These comparable results are not unexpected, as both tracer-gas mixtures had comparable physical properties. The Carbogen gas is similar to ambient air with an elevated CO_2_ concentration of 6%, while the ^81m^Kr-gas concentration was only 1–2% with the remainder being ambient air.

In this study the upper airway models were filled with the tracer gas. When flow of the tracer gas was stopped NHF was introduced. This quasi-static setup allowed for comparable and reproducible experiments to be performed. This is in contrast to the condition in vivo, where clearance of dead space already starts before the end of expiratory flow. This was demonstrated when expiring through the TM while a cannula flow of 30 l/min was applied (Fig. 2*B*). Initially the model was always “filled” with exhaled CO_2_ (Fig. 2*A*), but the same CO_2_ was significantly cleared at the end of expiration when a cannula flow such as 30 l/min was present (Fig. 2*B*). Fresh gas from a cannula was observed within the model even as the expiratory flow rate decreased from that of the peak flow, due to the difference of dynamic pressure between the nozzle and the cannula flows. This highlights that the clearance of dead space can be significantly affected by the breathing pattern and that the static condition in the experiments did not include the effects of breathing, thus underestimating the speed and effectiveness of the clearance.

In the 3D-printed UAM, only the radioactive ^81m^Kr-gas tracer-gas clearance protocol was followed, as the materials used to fabricate the model were not MWIR transparent. Similarly to the TM imaging analysis, the clearance rates were assessed in two adjoining ROIs, those being the anterior (UAM1) and posterior (UAM2) regions of the nasal cavities. The relationship between the clearance level and the NHF rate for the UAM was comparable to those obtained using the TM, with the clearance half-time of the ^81m^Kr-gas decreasing by increasing NHF rates. In both ROIs the change in clearance half-times was greater when the NHF was increased from 15 to 30 l/min than from 30 to 45 l/min. However, the clearance rates for both ROIs were calculated and were shown to have a linear response to an increase of the NHF rate (Fig. 5). An increased NHF rate of 1 l/min corresponded to ≈1.8 ml/s increase in the cleared nasal cavities volume in the UAM. In the deeper compartments beyond the soft palate (oropharynx, trachea), clearance half-times were greater than 1 s (see also Supplementary Video). These deeper regions of the conducting airways were not included in the data analyses, as clearance in these regions is more likely to be subjected to changes in flow restriction due to variability in the shape of the soft palate, the vocal cords, or the mouth opening. Consequently, clearance of the deeper regions of conducting airways has to be studied in vivo.

### 

#### Physiological and clinical implications.

It has been shown that NHF influences the gas exchange in the lungs with an increase in oxygen blood saturation (6, 17) and a reduction of arterial CO_2_ (17). A study on healthy volunteers revealed that the effects of NHF on ventilation was also dependent on whether the subject was awake or asleep: a reduction of tidal volume when awake and changes to minute ventilation when asleep (16). The authors of this paper speculated that the reduction of ventilation during sleep may be due to either a wash-out of anatomical dead space or a reduction in CO_2_ production.

It is known that during normal breathing at rest approximately one-third of tidal volume is rebreathed from the anatomical dead space (11). At the end of expiration, the dead space is filled with gas depleted in oxygen (15–16% compared with 21% in ambient air) and rich in CO_2_ (5–6% compared with 0.04% in ambient air). Therefore, an NHF-induced reduction of rebreathed CO_2_ volume should either decrease the tidal volume or respiratory rate to maintain the same alveolar ventilation. All of these conditions could eventually improve gas exchange. Improving the gas exchange through the reduction of dead space may either affect arterial blood gases or reduce minute ventilation, with a potential reduction in the work of breathing. Our data are limited to the dead space in the nasal cavities, but supports both the conclusions of Mundel et al. (16), that the reduction of dead space is the primary mechanism of decreased tidal volume with minute ventilation during sleep, and of Bräunlich et al. (3) on the reduction of the respiratory rate and minute ventilation. Reduction of respiratory rate, either through a decrease in rebreathing or through pressure effects as previously described in detail (16), may lead to a further reduction of dead space, as shown by the strong time dependence of clearance in this study. We speculate that a reduction in the respiratory rate may improve clearance by NHF therapy. Even in the absence of an end-expiratory pause, the slower respiration rate may lead to a more efficient clearance of dead space and a reduction of rebreathing.

The ratio of dead space to tidal volume (V_D_/V_T_) increases during shallow breathing, which in turn requires an increase of breathing frequency to maintain an adequate level of alveolar ventilation. Physiological dead space can be significantly increased in conditions like emphysema in chronic obstructive pulmonary disease (COPD) or pulmonary embolism by the elevated alveolar dead-space volume (11) that would lead to a high V_D_/V_T_ ratio, or in acute respiratory distress syndrome (ARDS) (9), which is associated with higher mortality. In these cases, even a small reduction of dead space would lead to a relatively high increase in alveolar volume. In this study the ROIs were limited to the nasal cavities, which includes between the nasal valve area and the soft palate, and have a combined volume of 55 cm^3^. Typical nasal cavity volumes of 40–50 cm^3^ were reported in healthy adults (23). Even this anatomical volume comprises at least one-third of the anatomical dead space (8) in adults and is significantly higher in children (19).^.^ Clearing this upper airway dead space could therefore be quite significant for patients who have elevated V_D_/V_T_ ratios. Similarly, the reduction of dead-space volume has been proposed as a mechanism that improves ventilation during purse-lip breathing, through forcing the flow to be unidirectional and bypassing the nasal cavities when exhaling (8).

The effectiveness of clearing the nasal cavities in the UAM (Fig. 5) has a linear positive dependency with NHF treatment. Therefore, the nasal cavity clearance level rises with increasing NHF rates, with every 1 l/min NHF increase leading to a 1.8 ml/s increased clearance in the nasal cavities. Independent of the variations between the geometries of the two upper airway models, the different tracer-gas properties, and imaging techniques used, all the results demonstrated very similar clearance levels during changes in the NHF rates. This further contributes to the notion that clearance of the dead space, especially within the nasal cavities, is strongly affected by the NHF rates. The size of the cannula may also have an influence on dead-space clearance due to the higher velocity of gas for a given flow. Smaller cannula may lead to more efficient dead-space clearance due also to more space, and hence leak, around the cannula. However, Mundel et al. (16) reported that the use of larger cannula leads to higher expiratory pressures, which may potentially reduce the respiratory rate and increase the tidal volume during wakefulness; this may, in turn, improve the efficiency of clearance and alveolar ventilation.

This study has investigated dead-space clearance under a quasi-static breathing condition, which occurs in the period between expiration and inspiration. At this stage of the breathing cycle the flow rates are reduced from low to no flow, shortly before reversing direction. During a normal breathing cycle this may take between 0.5 and 1.0 s, which would allow sufficient time for a tracer gas to be washed-out, based on the experimentally determined clearance half-times in the airway models used in this study (Fig. 5 and Tables 1–3). The quasi-static experimental condition resulted in an underestimation of the level of total clearance by NHF therapy, but allowed the clearance rates of these tracer gases to be studied without the added complexity of respiration.

Overall, the clearance profiles in the TM and UAM experiments exhibited similar NHF dependencies and this may indicate that deviations in the upper airway anatomy may not significantly modify the dead-space clearance characteristics using NHF in different subjects who share similar nasal cavity volumes. However, besides flow, the volume of the nasal cavities is an important parameter in describing NHF-induced clearance rates.

#### Strengths and limitations.

There are two key strengths in the current study. The first is the evaluation of upper airway dead-space clearance in two very different models of the upper airways: *1*) a very simple airway model, with the geometry limited to a tube with a streamlined nozzle to represent the narrow nasal valve area; and *2*) a more realistic model based on CT scans of a healthy individual, which encapsulated all the anatomical complexities of the upper airways. This allowed a direct comparison between the two models using the same imaging equipment. The results showed that both models responded in a similar manner: an increase of the NHF rate improved the clearance of dead space. This was demonstrated by the reduction of clearance half-times. Use of both models revealed that the anterior compartments cleared prior to the posterior sections. This adds weight to the argument that the geometry and dimension of the nasal cavities have less of an impact on dead-space clearance than the NHF rates. Clearance in the simple TM was only twice as fast as the very complex geometry of the UAM. The clearance of dead space by NHF increases linearly with an increase of flow, which is of clinical significance for the administration of NHF therapy.

The second key strength is that the upper airway clearance has been investigated using two distinct imaging modalities that use different tracer gases. Visualization and analysis of CO_2_ using MWIR transmission spectroscopy provided high temporal and spatial resolution for studying the clearance rates in a model with a simple geometry. Gamma imaging of ^81m^Kr-gas also produced comparable clearance rates in the same simple geometry model, as well as in a more complex but realistic upper airway model. Both methods and models produced comparable results that demonstrated the same clearance dynamics with increasing the NHF rates. The gamma ray imaging of ^81m^Kr-gas is of particular importance as this technique can be implemented for in vivo experiments.

There are several limitations in this study. The main drawback is that all experiments were performed with in vitro models, and only static clearance rates in the absence of breathing were quantified. The clearance responses to a range of tidal volumes and breathing patterns were not investigated during this study. The addition of breathing will only accelerate the clearance of the tracer gas from dead space; therefore, the results presented in this study underestimated the clearance levels. It was decided to limit the scope of this study to allow the accurate quantification of the NHF clearance rates in a simplified but repeatable protocol. Moreover, the effects of an open mouth, position of the soft palate, vocal cords, and the effects of changing the nasal prong positions were also not investigated. The analyses of the ROIs in the UAM were limited to the nasal cavity areas. The experiments with the two imaging methods did not produce identical results in TM2, due mainly to different acquisition rates, but demonstrated similar time and flow dependencies.

In summary, this study has shown effective clearance of the tracer gas, demonstrating similar dynamic characteristics despite the very different geometries of the upper airway models. The clearance is linearly related to the NHF rate with an anterior portion of the nasal cavities clearing faster than the posterior portion. We conclude that clearance of the nasal component of the anatomical dead-space with NHF therapy is a rapid process, which may significantly reduce CO_2_ rebreathing.

## GRANTS

The study was supported by a research grant from Fisher and Paykel Healthcare, Auckland, New Zealand.

## DISCLOSURES

W. Möller received research grants from Pari GmbH, Starnberg, Germany for studying nasal aerosolized drug delivery and from Fisher and Paykel Healthcare, Auckland, New Zealand, for studying the role of nasal high flow in dead space clearance. S. Feng and S. Tatkov are employees of Fisher and Paykel Healthcare, Auckland, New Zealand. All other authors declare no conflicts of interest.

## AUTHOR CONTRIBUTIONS

Author contributions: W.M., S.F., P.B., G.M., O.E., O.S., and S.T. conception and design of research; W.M., G.C., S.F., P.B., G.M., and S.T. performed experiments; W.M., G.C., and S.F. analyzed data; W.M., S.F., P.B., G.M., O.E., O.S., and S.T. interpreted results of experiments; W.M., G.C., and S.F. prepared figures; W.M., S.F., and S.T. drafted manuscript; W.M., S.F., P.B., G.M., O.E., O.S., and S.T. edited and revised manuscript; W.M., G.C., S.F., P.B., G.M., O.E., O.S., and S.T. approved final version of manuscript.

## Supplementary Material

Video S1
